# A Single‐Dose mRNA Vaccine Employing Porous Silica Nanoparticles Induces Robust Immune Responses Against the Zika Virus

**DOI:** 10.1002/advs.202404590

**Published:** 2024-07-15

**Authors:** Hojeong Shin, Seounghun Kang, Cheolhee Won, Dal‐Hee Min

**Affiliations:** ^1^ Department of Chemistry Seoul National University Seoul 08826 Republic of Korea; ^2^ Institute of Biotherapeutics Convergence Technology Lemonex Inc. Seoul 06683 Republic of Korea

**Keywords:** local delivery, mRNA vaccine, porous silica nanoparticles, virus vaccine, zika virus

## Abstract

Recently, lipid nanoparticles (LNPs)‐based mRNA delivery has been approved by the FDA for SARS‐CoV‐2 vaccines. However, there are still considerable points for improvement in LNPs. Especially, local administration of LNPs‐formulated mRNA can cause off‐target translation of mRNA in distal organs which can induce unintended adverse effects. With the hypothesis that large and rigid nanoparticles can be applied to enhance retention of nanoparticles at the injection site, a polyethyleneimine (PEI)‐coated porous silica nanoparticles (PPSNs)‐based mRNA delivery platform is designed. PPSNs not only facilitate localized translation of mRNA at the site of injection but also prolonged protein expression. It is further demonstrated that the development of a highly efficacious Zika virus (ZIKV) vaccine using mRNA encoding full‐length ZIKV pre‐membrane (prM) and envelope (E) protein delivered by PPSNs. The ZIKV prME mRNA‐loaded PPSNs vaccine elicits robust immune responses, including high levels of neutralizing antibodies and ZIKV E‐specific T cell responses in C57BL/6 mice. Moreover, a single injection of prME‐PPSNs vaccine provided complete protection against the ZIKV challenge in mice.

## Graphical abstract

Although ZIKV has been found to induce severe neuropathology, there are no vaccines or medications yet. Here, the development of a highly efficacious ZIKV vaccine is reported using mRNA encoding full‐length ZIKV pre‐membrane and envelope protein delivered by porous silica nanoparticles that facilitate localized translation of mRNA at the site of injection and prolonged protein expression.

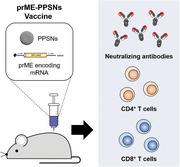

## 1 Introduction

In recent years, the mRNA vaccine platform has advanced rapidly.^[^ ^1^, ^2^, ^3^ ^]^ mRNA‐based therapeutics have several benefits over plasmid DNA‐mediated gene therapy: (1) mRNA molecules do not require access to the nucleus; (2) they have no potential risk for genomic integration; and (3) mRNA drugs can be developed quickly. However, mRNA therapeutics possess some limitations. The intrinsic negative charge and large size of mRNA molecules hinder cell membrane penetration. Furthermore, mRNA molecules are highly susceptible to degradation by ribonucleases (RNases), which makes mRNA delivery challenging. Thus, an appropriate delivery vehicle is needed to protect mRNA against enzyme digestion and enhance cellular uptake.^[^ ^4^, ^5^ ^]^

Lipid nanoparticles (LNPs) are one of the most frequently used delivery vehicles in transportation of mRNA in vivo.^[^ ^4^, ^6^ ^]^ Intramuscularly administered LNPs have delivered mRNA to protect against COVID‐19. Despite this encouraging progress, there are still considerable points for improvement. Especially, the local injection of LNPs‐formulated mRNA causes off‐target expression of transgene in distant tissues because of passive diffusion of LNPs.^[^ ^7^, ^8^, ^9^ ^]^ Off‐target translation of mRNA and systemic spread of LNPs can induce unintended adverse effects.^[^ ^10^, ^11^, ^12^ ^]^ It makes them extremely challenging to locally deliver mRNA to the injection site. Therefore, an alternative strategy that addresses this drawback is required to maximize the potential of mRNA therapeutics.

Previous studies have demonstrated that particle size and surface functionality play an essential role in local retention.^[^ ^13^, ^14^, ^15^ ^]^ It is reported that larger nanoparticles have longer retention in the tissues and nanoparticles smaller than 200 nm transport from the injection site to lymph nodes through the lymphatic system.^[^ ^16^, ^17^, ^18^ ^]^ Di et al. have demonstrated larger LNPs accumulate and achieve higher mRNA translation in the injection site compared to small LNPs.^[^ ^19^ ^]^ Additionally, it has also been shown that rigid nanoparticles have a longer retention duration at the injection site than soft nanoparticles.^[^ ^17^, ^20^ ^]^ Therefore, we hypothesized that large‐sized rigid nanoparticles may take advantage of a lower migration through the interstitial space, allowing higher accumulation at the site of injection. Among various rigid nanoparticles, we chose porous silica nanoparticles (PSNs) because of the following: (1) PSNs can be simply produced with tunable particle sizes; (2) they are biodegradable, which allows reduced toxicity and easy excretion; and (3) facile surface modification that enables higher loading efficiency and the ability to protect nucleic acids from nuclease degradation.^[^ ^5^, ^21^ ^]^

Herein, we report the polyethyleneimine (PEI)‐coated PSNs (PPSNs)‐based mRNA delivery platform to specifically deliver mRNA into the site of injection, in which target proteins can be stably expressed. PPSNs achieved not only localized protein expression at the injection site but also prolonged mRNA translation in vivo. Therefore, concerns regarding off‐target effects and accompanying systemic toxicities can be reduced. We further demonstrated that the PPSNs‐based mRNA vaccine composed of mRNA encoding pre‐membrane (prM) and envelope (E) protein of Zika virus (ZIKV) is highly immunogenic in eliciting robust humoral and cellular responses against ZIKV. Moreover, the PPSNs‐based vaccine was able to protect mice against lethal ZIKV challenges.

## 2 Results

### 2.1 Preparation and Characterization of PPSNs

**Figure 1:**
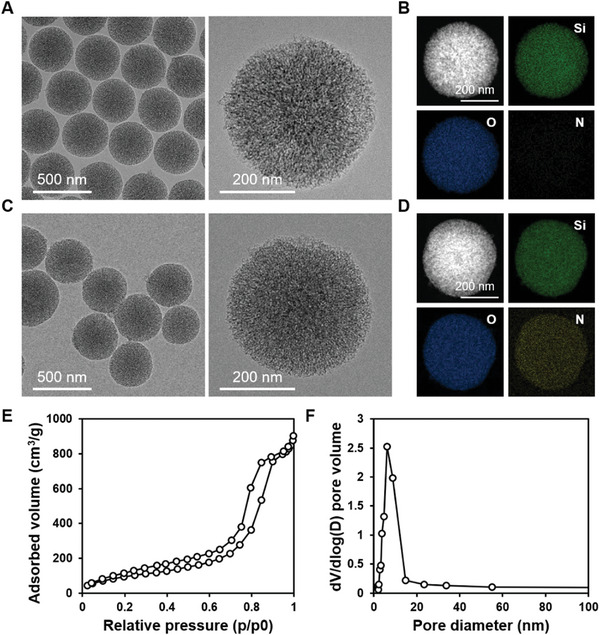
Characterization of PPSNs. A) A representative TEM image of bare PSNs (left) and an individual bare PSN (right). B) TEM‐EDS elemental analysis of bare PSN. C) A representative TEM image of PPSNs (left) and an individual PPSN (right). D) TEM‐EDS elemental analysis of PPSN, confirming the presence of Si, O, and N. E) N_2_ adsorption‐desorption isotherms and (F) pore size distribution of PPSNs.

In accordance with our previous investigation,^[^ ^15^, ^22^ ^]^ bare PSNs were synthesized using CTAB as structure‐directing agents and TMOS as a silica precursor. In order to facilitate efficient mRNA loading, a branched PEI with a molecular weight of 1.8 kDa was utilized to modify the surface of the PSNs and confer a positive charge. Transmission electron microscopy (TEM) showed the successful synthesis of bare PSNs (**Figure** **1**A) and PPSNs with an average nanoparticle size of about 350 nm (Figure 1C). Energy‐dispersive spectrometry (EDS) elemental mapping results showed that the bare PSNs did not exhibit any detectable nitrogen (N) signal (Figure 1B), while the PPSNs exhibited a strong N signal, indicating the successful incorporation of PEI onto the surface (Figure 1D). The N_2_ adsorption‐desorption isotherm (Figure 1E) and the pore size distribution curve (Figure 1F) indicated a pore size centered at about 6.08 nm. mRNA loading capacity was evaluated using a gel retardation assay, which revealed that 1 µg of PPSNs was required to load 0.5 µg of mRNA (Figure S1A, Supporting Information). The zeta potential of PPSNs decreased from 43.1 mV to 5.21 mV when mRNA molecules were loaded (Figure S1B, Supporting Information). We proceeded to evaluate the cytotoxicity of PPSNs using the CCK‐8 assay (Figure S2, Supporting Information). In order to assess the potential toxicity of PPSNs on cells, we incubated the myoblast cell line, C2C12, with varying concentrations of PPSNs and measured their viability. Our results demonstrated that there was no significant difference in the viability of C2C12 cells when exposed to PPSN concentrations up to 125 µg mL^−1^ after 24 h. Furthermore, with increasing exposure time, the cell viability remained at 94.4% after 72 h, even at a relatively high concentration of 62.5 µg mL^−1^.

**Figure 2:**
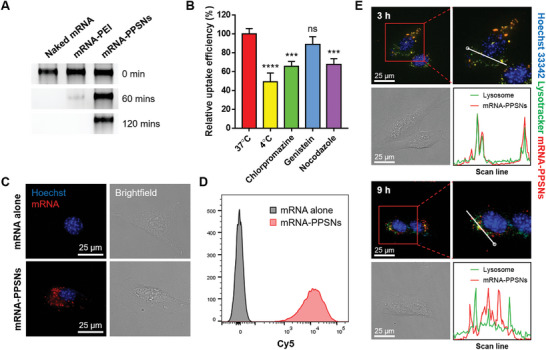
Cellular internalization and endosomal escape of mRNA‐loaded PPSNs. A) mRNA protection assay from RNase‐mediated degradation. Lane 1: naked mRNA, Lane 2: mRNA‐PEI polyplexes, Lane 3: mRNA‐PPSNs complexes. The data demonstrate that mRNA loaded onto PPSNs remains intact even after incubation with RNase for 2 h at 37 °C. B) Cellular internalization efficiency of mRNA‐PPSNs complexes in presence of endocytosis inhibitors. The median fluorescence intensity (MFI) values of the cells were normalized to the MFI of the control group, which was incubated at 37 °C without any endocytosis inhibitors. Results are shown as mean with SD (*n* = 3). Statistical significance was determined using one‐way ANOVA. ns: not‐significant, ***P < 0.001, and ****P < 0.0001. C) Representative images of C2C12 cells treated with mRNA alone or mRNA‐PPSNs. D) In vitro uptake efficiency of Cy5‐labeled mRNA by C2C12 cells measured by flow cytometry. E) Endosomal escape of mRNA‐PPSNs complexes. Fluorescent visualization of C2C12 cells treated with mRNA‐PPSNs for 3 and 9 h; nuclei (blue), endosomes (green), and mRNA (red). Relative fluorescence intensities of lysotracker and mRNA‐PPSNs were measured along with a line scan profile in living cells.

To evaluate the capacity of PPSNs to protect mRNA payload against nuclease‐mediated degradation, we conducted a RNase protection assay (**Figure** **2**A). Equal amounts of mRNA were loaded onto PPSNs or naïve PEI, and these complexes were subjected to treatment with RNase for up to 2 h. Subsequently, heparin was used to dissociate the mRNA from the particles, and the samples were analyzed using a gel retardation assay. Our findings demonstrated that a significant portion of the mRNA loaded onto PPSNs remained intact even after a 2‐hour incubation with RNase, in contrast to mRNA associated with PEI, which underwent complete degradation. These results indicate that PPSNs effectively shield mRNA from RNase‐mediated degradation, offering protection against enzymatic degradation.

### 2.2 Cellular Uptake and Endosomal Escape of PPSNs

Having confirmed the successful preparation of PPSNs, we next sought to determine cellular uptake and endosomal escape of PPSNs, which are critical for their biological applications. First, we investigated whether PPSNs enable mRNA to be internalized into cells. When C2C12 cells were treated with Cy5 conjugated mRNA encapsulated into PPSNs, red fluorescence was significantly shown in the cytoplasm (Figure 2C). In contrast, no fluorescence appeared in the cells treated with Cy5‐labeled mRNA alone. The results from the flow cytometry was highly correlative with fluorescence imaging (Figure 2D). Taken together, the data indicated that the present mRNA‐PPSNs complexes were readily uptaken to the C2C12 cells, suggesting its potential for cytosolic delivery of mRNA molecules.

To elucidate the endocytosis pathway of PPSNs, we investigated their cellular uptake in C2C12 cells. We observed that the cellular uptake of PPSNs was markedly reduced by 49.2% when cells were incubated at 4 °C, indicating that PPSN endocytosis is an energy‐dependent process. To determine the specific endocytosis pathway utilized by PPSNs, we treated C2C12 cells with Cy5‐mRNA‐loaded PPSNs along with chlorpromazine, genistein, or nocodazole, which respectively inhibit clathrin‐associated endocytosis, caveolae‐mediated endocytosis, and macropinocytosis (Figure 2B). Our results indicate that PPSNs are internalized through both clathrin‐dependent endocytosis and macropinocytosis, as evidenced by the significant reduction in Cy5‐mRNA‐PPSN uptake upon treatment with chlorpromazine and nocodazole compared to the control group (*P* = 0.0005 and 0.0007, respectively). On the other hand, genistein did not significantly inhibit particle internalization (*P* = 0.2234).

Drug delivery carriers should overcome the barrier associated with the endolysosomal pathway because nanoparticles and cargos can be degraded in endosomes because of the acidic condition and enzymes. PEI is considered to escape endosomes by the proton sponge approach, whereby PEI possesses a proton buffering capacity that results in rupturing endosomes by osmotic swelling. After 3 and 9 h of incubation with Cy5‐mRNA‐encapsulated PPSNs, C2C12 cells were stained with Hoechst 33 342 and LysoTracker Green. The orangish signal originated from the colocalization of the mRNA‐loaded PPSNs with the endosomes was shown at 3 h and after another 6 h, some red signal from mRNA was observed outside of the endosomes indicating endosomal escape (Figure 2E). Taken together, these data suggest that PPSNs enable cytosolic deliver of mRNA payloads and are able to successfully escape the endosomal compartment.

### 2.3 PPSNs have Favorable In Vitro and In Vivo Characteristics as mRNA Delivery Vehicles

**Figure 3:**
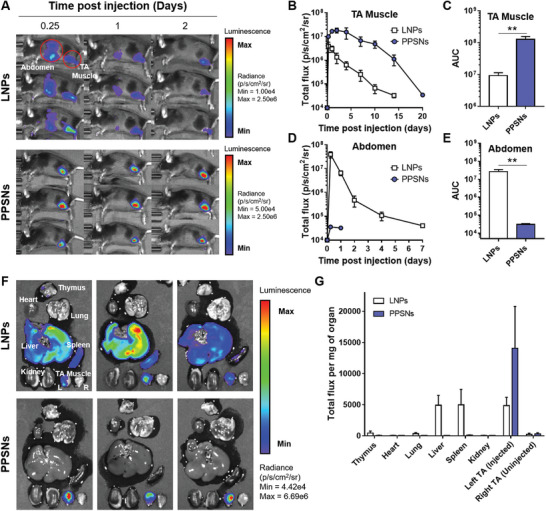
In vivo expression pattern of PPSNs‐encapsulated FLuc mRNA. A) Representative IVIS images of C57BL/6 mice injected intramuscularly with 10 µg of FLuc mRNA encapsulated by LNPs or PPSNs. B–E) Time course of bioluminescent signal quantification in (B) TA muscle and (D) abdomen. Area under the curve (AUC) analysis of bioluminescence measured from (C) TA muscle and (E) abdomen of each treatment. Data are presented as mean with SD (*n* = 3). Statistical significance was determined using Student's *t*‐test. **P < 0.01. F) Ex vivo bioluminescence imaging of tissues was performed 6 h post‐injection in C57BL/6 mice. The mice were injected with 10 µg of FLuc mRNA encapsulated into LNPs or PPSNs in the TA muscle of the left hind limb. G) Quantification of the total flux per miligram of each organ. Data are presented as mean with SD (*n* = 3).

Next, we evaluated the protein expression efficiency, kinetics, and biodistribution upon intramuscular injection of mRNA‐PPSNs complexes using mRNA encoding firefly luciferase (FLuc). A comparison was made using LNPs formulated with ALC‐0315. LNPs exhibited an average diameter of 105.6 ± 2.3 nm and the zeta potential of 2.18 mV. The encapsulation efficiency was measured at a 88.3%. PPSNs or LNPs‐formulated FLuc mRNA was injected into the tibialis anterior (TA) muscle of mice and mRNA translation was monitored using in vivo bioluminescence imaging (**Figure** **3**A). The results revealed that PPSNs outperformed LNPs in terms of both the level and duration of mRNA translation at the injection site (Figure 3B). The bioluminescent signal persisted for ≈ 14 days in mice receiving FLuc‐LNPs injections, whereas mice receiving FLuc‐PPSNs injections exhibited a signal that lasted up to 20 days. The area under the curve (AUC) value from the injection site was 13.8‐fold higher in the FLuc‐PPSNs group compared to the FLuc‐LNPs group (Figure 3C). Additionally, a significant bioluminescent signal was observed in the abdomen of FLuc‐LNPs‐injected mice, whereas mice injected with FLuc‐PPSNs showed minimal off‐target signal, indicating no notable systemic spread of PPSNs in contrast to LNPs (Figure 3D). The AUC value of the signal from the abdomen was 845‐fold higher in FLuc‐LNPs group than in FLuc‐PPSNs group (Figure 3E). When the AUC values for the muscle and abdomen were combined, the FLuc‐PPSNs group exhibited 3.57‐fold higher values compared to the FLuc‐LNPs group. As controls, when the carriers were administered without the mRNA payload, no detectable signals were observed in the body (Figure S3, Supporting Information).

Furthermore, to assess the total luminescence signal per organ, the major organs were imaged ex vivo. FLuc mRNA was injected into the TA muscle using either LNPs or PPSNs and major organs were collected 6 h after injection (Figure 3F). The mRNA delivered by PPSNs primarily translated in the injected TA muscle, with negligible signal detected in other organs. Conversely, robust signals were detected not only in the muscle but also in the liver and spleen of the FLuc‐LNPs injected group. The quantification of the luminescence signal further confirmed the distinct biodistribution patterns between LNPs and PPSNs (Figure 3G). These distinct biodistribution profiles between FLuc‐LNPs and FLuc‐PPSNs were likely attributable to the different physicochemical properties of the delivery vehicles. Overall, these findings demonstrate that the PPSNs‐based mRNA delivery platform exhibits a promising biodistribution profile without hepatic accumulation and simultaneously achieves efficient and sustained mRNA translation at the site of injection.

Next, we conducted an assessment of the biodistribution pattern of PPSNs. To investigate the distribution of the mRNA loaded onto PPSNs, we labeled mRNA with a fluorescent dye and administered mRNA‐PPSNs complexes via intramuscular injections. Subsequently, we performed ex vivo fluorescence imaging to examine the distribution of the mRNA in various organs (Figure S4, Supporting Information). Our findings revealed that the signals originating from the injected mRNA‐loaded PPSNs were predominantly localized within the TA muscle, consistent with the bioluminescence imaging results. Furthermore, we observed signals originating from the mRNA‐loaded PPSNs in the axillary, iliac, and inguinal lymph nodes, indicating their ability to traverse beyond the injection site and enter the lymphatic system. Notably, the signal intensity for the mRNA‐loaded PPSNs increased over time, with the strongest signals observed on day 5. This phenomenon could potentially be attributed to the size of the particles, as the particles larger than ≈100 nm in diameter are known to show reduced passive drainage. Thus, we speculate that these particles may have been taken up by muscle‐resident antigen‐presenting cells, which subsequently migrated to the lymph nodes, resulting in the observed signal in lymph nodes.

### 2.4 ZIKV prME‐PPSNs Vaccine Induces Antibody Responses with Neutralizing Activities Against ZIKV

As shown above, PPSNs outperformed LNPs with regard to protein expression level and duration. Therefore, we next sought to examine the immune response that developed toward the ZIKV antigen. To induce potent immune responses against ZIKV, we designed mRNA‐encoded prME glycoproteins of ZIKV SPH/2015 containing the signal sequence from the Japanese encephalitis virus (JEV), which could increase the subviral particle (SVP) release (Figure S5A, Supporting Information). The expression of the ZIKV E protein was confirmed by analyzing both cell lysates and supernatant collected from HEK293T cells 24 h after transfection. A non‐transfected group served as the negative control, while recombinant ZIKV E antigen was used as the positive control. Additionally, a control group transfected with FLuc mRNA was included. Western blot using an anti‐flavivirus E protein antibody revealed distinct bands in the prME mRNA‐transfected cells and the ZIKV E antigen group, while no bands were detected in the other control groups (Figure S5B, Supporting Information). In addition to western blot analysis, we employed immunofluorescence staining to visualize the cytosolic expression of the E protein. To establish ZIKV‐infected cells as a positive control, Huh‐7 cells, a mammalian cell line susceptible to ZIKV, were included in the experiments. The immunofluorescence staining images provided clear evidence of cytosolic expression of the E protein in prME mRNA‐transfected Huh‐7 cells (Figure S5C, Supporting Information).

**Figure 4:**
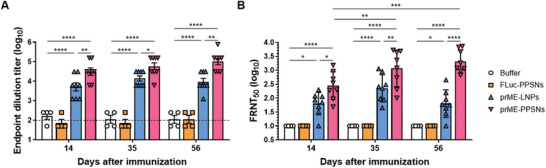
prME‐PPSNs vaccines are immunogenic in mice. C57BL/6 mice were intramuscularly immunized with buffer (*n* = 4), FLuc‐PPSNs (*n* = 4), ZIKV prME mRNA‐LNPs (*n* = 8), or ZIKV prME mRNA‐PPSNs (*n* = 8). A) ZIKV E‐protein specific IgG was analyzed by ELISA at day 14, 35, and 56. Data are presented as mean with SD. Statistical significance was determined using two‐way ANOVA. *P < 0.05, **P < 0.01, and ****P < 0.0001. Dotted line indicates the cut‐off value. B) Neutralizing antibody activity was determined by FRNT assay using ZIKV Brazil/16 321 at day 14, 35, and 56. Data are presented as mean with SD. Statistical significance was determined using two‐way ANOVA. *P < 0.05, **P < 0.01, ***P < 0.001, and ****P < 0.0001.

Next, we proceeded to investigate the capacity of ZIKV prME mRNA loaded onto PPSNs to induce humoral immune responses in mice. We analyzed the ZIKV E‐specific antibody responses using IgG ELISA in C57BL/6 mice following vaccination (**Figure** **4**A). The mice received a single intramuscular injection of buffer, FLuc‐PPSNs, prME‐LNPs, or prME‐PPSNs, with FLuc or prME mRNA at a dose of 15 µg per mouse. ZIKV E protein‐specific IgG titers in the serum were measured on days 14, 35, and 56. Immunization with ZIKV prME‐PPSNs elicited robust humoral responses against ZIKV. The levels of E protein‐specific IgG in the serum of prME‐PPSNs immunized mice increased rapidly and remained consistently high, with an endpoint titer of 180,000 on day 56. In contrast, the serum levels of E protein‐specific IgG in prME‐LNPs vaccinated mice peaked on day 35 (endpoint titer of approximately 18,000) and subsequently declined.

Additionally, we assessed the ability of antibodies from PPSNs‐immunized mice to neutralize ZIKV and confirmed a high neutralizing activity (Figure 4B; Figure S6, Supporting Information). Anti‐ZIKV neutralizing antibodies were measured using a focus reduction neutralization test (FRNT) with the ZIKV Brazil/16 321. Mice immunized with ZIKV prME‐PPSNs exhibited significantly higher levels of neutralizing antibodies, consistent with the IgG titers. The mean FRNT_50_ titers against ZIKV Brazil/16 321 in prME‐PPSNs immunized mice reached approximately 2,300 on day 35 and increased to around 3,000 by day 56. In contrast, the ability to neutralize ZIKV Brazil/16 321 was markedly reduced in prME‐LNPs vaccinated mice. The mean FRNT_50_ titer in prME‐LNPs vaccinated mice peaked at approximately 400 on day 35 and declined to around 100 by day 56. Collectively, these findings demonstrate the capacity of PPSNs to induce potent humoral immune responses against ZIKV, highlighting their potential as an effective vaccine strategy.

### 2.5 ZIKV prME‐PPSNs Vaccine Elicits ZIKV E Protein‐Specific T Cell Responses

Given the importance of T cell responses in controlling ZIKV infection,^[^ ^23^, ^24^, ^25^, ^26^ ^]^ we measured the ability of ZIKV specific T cell populations from immunized mice to secrete IFN‐γ, TNF‐α, and IL‐2 in response to ZIKV E protein stimulation. C57BL/6 mice were immunized via intramuscular injection with buffer, FLuc‐PPSNs, prME‐LNPs, or prME‐PPSNs, and CD4^+^ and CD8^+^ T cell responses were evaluated on day 14 post‐vaccination. Intracellular cytokine staining was utilized to assess the population of ZIKV E‐specific T cells and determine the specificity and pattern of cytokine production from ZIKV‐specific CD4^+^ and CD8^+^ T cells.

**Figure 5:**
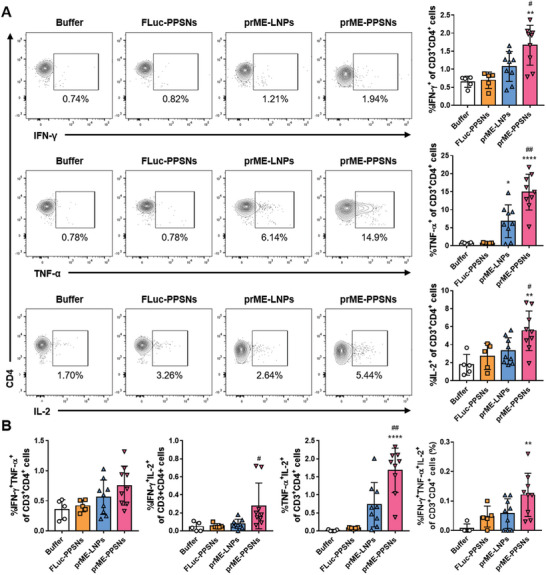
Induction of CD4^+^ T cell responses by prME‐PPSNs. C57BL/6 mice received intramuscular injection of buffer (*n* = 5), FLuc‐PPSNs (*n* = 5), ZIKV prME mRNA‐LNPs (*n* = 9) or ZIKV prME mRNA‐PPSNs (*n* = 9). Splenocytes were isolated on day 14 for analysis of splenic antigen‐specific CD4+ T cells. After stimulation with ZIKV E protein for 6 h, the frequency of IFN‐γ^+^, TNF‐α^+^, and IL‐2^+^ CD4^+^ T cells was measured using intracellular cytokine staining. A) Representative dot plots and quantitative data demonstrate the gating procedure for analyzing single cytokine production in CD4^+^ T cells. Quantitative analysis of B) double and triple cytokine producers in CD4^+^ T cells in splenocytes. Data are presented as mean with SD. Statistical significance was determined using one‐way ANOVA. Asterisks (*P < 0.05, **P < 0.01, and ****P < 0.0001) indicate the comparison of prME‐LNPs and prME‐PPSNs to the buffer group. The differences between prME‐LNPs and prME‐PPSNs are indicated by hashtags (^#^ *P* < 0.05 and ^##^ *P* < 0.01). Not‐significant p‐values are not indicated.

**Figure 6:**
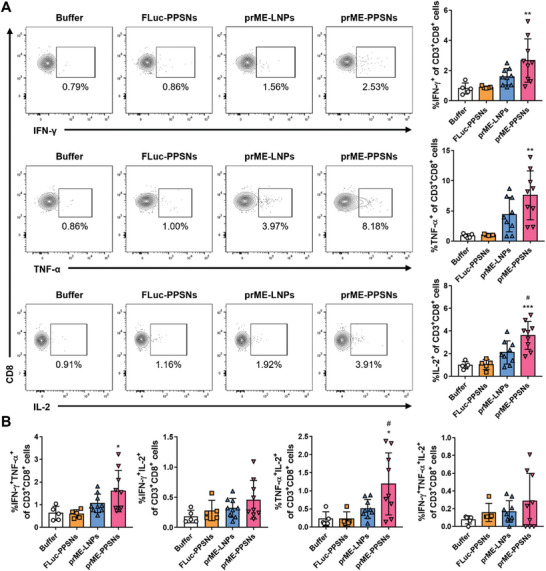
Induction of CD8^+^ T cell responses by prME‐PPSNs. C57BL/6 mice received intramuscular injection of buffer (*n* = 5), FLuc‐PPSNs (*n* = 5), ZIKV prME mRNA‐LNPs (*n* = 9) or ZIKV prME mRNA‐PPSNs (*n* = 9). Splenocytes were isolated on day 14 for analysis of splenic antigen‐specific CD8+ T cells. After stimulation with ZIKV E protein for 6 h, the frequency of IFN‐γ^+^, TNF‐α^+^, and IL‐2^+^ CD8^+^ T cells was measured using intracellular cytokine staining. A) Representative dot plots and quantitative data demonstrate the gating procedure for analyzing single cytokine production in CD8^+^ T cells. Quantitative analysis of B) double and triple cytokine producers in CD8^+^ T cells in splenocytes. Data are presented as mean with SD. Statistical significance was determined using one‐way ANOVA. Asterisks (*P < 0.05, **P < 0.01, and ***P < 0.001) indicate the comparison of prME‐LNPs and prME‐PPSNs to the buffer group. The differences between prME‐LNPs and prME‐PPSNs are indicated by hashtags (^#^ *P* < 0.05). Not‐significant p‐values are not indicated.

The findings revealed distinct cytokine production profiles between the CD4^+^ and CD8^+^ subsets, with TNF‐α and IL‐2 responses being dominant in CD4^+^ subsets (**Figure** **5**A) and IFN‐γ responses being dominant in CD8^+^ T cells (**Figure** **6**A). Additionally, mice immunized with prME‐PPSNs exhibited higher levels of cytokine secretion in CD4^+^ and CD8^+^ T cells compared to those immunized with prME‐LNPs. Specifically, the frequencies of IFN‐γ, TNF‐α, and IL‐2‐positive CD4^+^ T cells induced by splenocytes of prME‐PPSNs immunized mice were significantly higher than those of prME‐LNPs immunized mice (*P* = 0.0288, 0.0010, and 0.0460, respectively). Moreover, the prME‐PPSNs immunized group demonstrated significantly higher IL‐2 responses in CD8^+^ T cells compared to the prME‐LNPs‐immunized group (*P* = 0.0133), indicating the favorable impact of PPSNs over LNPs on the outcome of the ZIKV‐specific CD4 immune response. While the cellular responses of immune splenocytes from prME‐LNPs vaccinated mice were generally elevated compared to buffer‐injected mice, the differences were not statistically significant. In the prME‐PPSNs group, ZIKV E‐specific responses exhibited a high degree of polyfunctionality, with 0.12% and 0.29% of responding CD4^+^ and CD8^+^ T cells, respectively, exhibiting three functions (Figures 5, 6). These findings highlight that the ZIKV prME mRNA‐PPSNs vaccine candidate induced antigen‐specific CD4^+^ and CD8^+^ T cell responses with significant polyfunctionality, underscoring its potential in controlling ZIKV infection.

We next evaluated the Th1/Th2 cytokine secretion profile of prME‐PPSNs vaccinated mice. Immunized mice were sacrificed 4 weeks after immunization with prME mRNA‐loaded PPSNs, and an ELISpot assay was conducted. As shown in Figure S7 (Supporting Information), a specific and statistically significant secretion of IFN‐γ and IL‐4 was observed in both prME‐PPSNs and prME‐LNPs vaccinated mice compared to vehicle treatment. However, the secretion of IFN‐γ and IL‐4 in splenocytes from the prME‐PPSNs immunized mice was significantly higher than in those from the prME‐LNPs immunized mice (*P* = 0.0424 and 0.0442, respectively). Plotting the results of IFN‐γ versus IL‐4 indicated the induction of a Th1‐biased cellular response by prME‐PPSNs.

### 2.6 prME‐PPSNs Vaccine Provides Complete Protection Against Lethal ZIKV Infection and a Favorable Safety Profile

**Figure 7:**
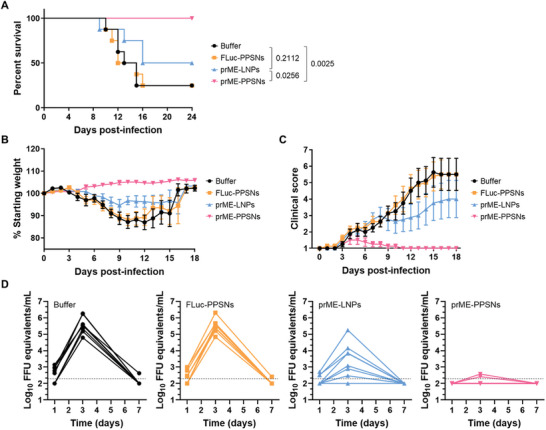
A single immunization of ZIKV prME‐PPSNs provides protection against lethal ZIKV challenge. C57BL/6 mice were immunized with buffer, FLuc‐PPSNs, ZIKV prME mRNA‐LNPs or ZIKV prME mRNA‐PPSNs (*n* = 8). At week 8, vaccinated C57BL/6 mice were administered 2 mg of anti‐INFAR‐1 blocking antibody and 1 day later challenged with 10^3^ FFU of ZIKV MR766. Animals were monitored for A) survival, B) weight loss, and C) clinical score. D) Serum was collected at 1, 3 and 7 days after viral challenge and analyzed for levels of ZIKV RNA. Dotted lines indicate the limit of detection. Statistical significance was determined using the Log‐rank test (A). Data are presented as mean with SD (B) and mean with SEM (C).

To evaluate the protective efficacy of the prME‐PPSNs vaccine against lethal ZIKV challenge, C57BL/6 mice were vaccinated intramuscularly with 15 µg of prME mRNA loaded onto PPSNs, prME mRNA encapsulated LNPs, FLuc mRNA loaded onto PPSNs, or buffer. At 56 days post‐immunization, the vaccinated mice were subcutaneously inoculated in the footpad with 10^3^ focus‐forming units (FFU) of the mouse‐adapted African genotype ZIKV strain MR 766. To render the mice susceptible to ZIKV infection, INFAR‐1 blocking antibody (MAR1‐5A3) was intraperitoneally injected one day before the viral challenge. Daily observations were made regarding weight loss, clinical score, and survival rate following the challenge (**Figure** **7**A–C).

Mice receiving prME‐PPSNs did not exhibit body weight reduction or severe clinical symptoms, while most of the buffer‐injected mice experienced significant weight loss and clinical signs. Additionally, all prME‐PPSNs immunized mice survived, in contrast to the buffer group and prME‐LNPs group, which showed survival rates of 25% and 50%, respectively. The prME‐PPSNs group demonstrated prolonged survival compared to the buffer or prME‐LNPs groups (*P* = 0.0025 and 0.0256, respectively). The FLuc‐PPSNs group showed no statistical difference compared to the buffer group, indicating that the protection against ZIKV in the prME‐PPSNs group is mainly originated from the expression of ZIKV antigen.

The protective efficacy of the prME‐PPSNs vaccination was further determined by measuring viremia via qRT‐PCR on days 1, 3 and 7 post‐challenge (Figure 7D,E). Mice receiving either prME‐PPSNs or prME‐LNPs vaccination exhibited significantly reduced viremia compared to those receiving buffer injections (evaluated by comparing AUC through one‐way ANOVA; *P* = 0.0072 and *P* < 0.0001, respectively). The control group displayed a peak viral RNA loads with a median of ≈ 5.56 log_10_ FFU equivalents mL^−1^ on day 3. Mice vaccinated with prME‐PPSNs demonstrated substantial protection from viremia, as 6 out of 8 mice exhibited undetectable viremia. In contrast, mice vaccinated with prME‐LNPs showed the presence of viral RNA in the blood on day 3, with a median peak of ≈ 3.44 log_10_ FFU equivalents mL^−1^, indicating that the prME‐PPSNs vaccine provides greater protection compared to the prME‐LNPs vaccine.

Furthermore, we assessed the protective effect of the mRNA vaccine using the Asian genotype ZIKV strain Brazil/16 321 (Figure S8, Supporting Information). As the Asian genotype ZIKV did not cause mortality in immunocompetent mice, even when sensitized with MAR1‐5A3,^[^ ^27^ ^]^ serum viral load comparisons were made between the buffer group and prME‐PPSNs immunized group. The buffer group consistently exhibited high viral loads (median peak of around 4.00 log_10_ FFU equivalents/mL) on day 3 after ZIKV inoculation, whereas 5 out of 6 prME‐PPSNs group showed absence of viral RNA in serum, indicating significant protection. These findings demonstrate that a single immunization with the prME‐PPSNs vaccine rapidly induces protection for a duration of 8 weeks against detectable viremia caused by various strains of ZIKV in mice.

### 2.7 In Vivo Toxicity Evaluation of prME‐PPSNs

Finally, we conducted an in vivo assessment of the safety profile of prME‐PPSNs by administering intramuscular injections to mice at the therapeutic dose (15 µg of mRNA and 30 µg of PPSNs), followed by blood analysis for a period of 7 days (Figure S9, Supporting Information). Both the buffer and prME‐PPSNs groups showed similar results in terms of toxicity evaluation based on blood tests, including blood chemistry and complete blood count. The serum parameters of treated mice, such as liver function markers (aspartate aminotransferase [AST] and alanine aminotransferase [ALT]) and kidney function indicators (blood urea nitrogen [BUN] and creatinine), were comparable to those of the control group. Moreover, chronic toxicity indicators (total protein level) and cell membrane damage biomarkers (lactate dehydrogenase [LDH]) exhibited similar trends. Complete blood panel analysis also revealed no significant differences in the counts of white blood cells, red blood cells, or platelets between the buffer and treatment groups at each time point after administration. These findings suggest that there is little evidence of systemic toxicity following prME‐PPSNs injection, which is consistent with the limited systemic spread observed. In addition, a histopathological study was conducted to evaluate the safety profile of the prME‐PPSNs vaccine. The major organs and TA muscle of mice vaccinated with prME‐PPSNs did not show any histopathological changes compared to the control group (Figure S10, Supporting Information).

Furthermore, our investigation extended to a comparative analysis of in vivo toxicity between prME‐PPSNs and prME‐LNPs, with a focus on evaluating their acute toxicity effects. Mice were administered high doses of prME‐PPSNs (5 and 50 mg kg^−1^), prME‐LNPs (5 mg kg^−1^), or buffer as a negative control. Subsequent serum sample analyses were conducted, and the results revealed a significant difference (Figure S11, Supporting Information). Specifically, mice treated with prME‐LNPs exhibited notably higher serum levels of ALT and AST in comparison to buffer‐treated mice. In contrast, mice treated with prME‐PPSNs did not display elevated levels of any liver toxicity markers measured in comparison to PBS‐treated mice. Collectively, a comprehensive series of in vivo studies demonstrated the high effectiveness and safety of the prME‐PPSNs vaccine.

## 3 Discussion and Conclusion

In our study, we present an efficient mRNA vaccine candidate against ZIKV utilizing PPSNs as a delivery platform. PPSNs possess physicochemical properties that enable localized protein expression at the injection site and sustained mRNA translation. Once taken up by surrounding cells, the mRNA molecules are released from the PPSNs, leading to the synthesis of antigens. Our findings demonstrate that a single immunization with 15 µg mRNA‐loaded PPSNs induces robust humoral and cellular immune responses, conferring protection against both lethal African genotype and non‐lethal Asian genotype ZIKV infections in C57BL/6 mice. The resulting anti‐ZIKV E sera were able to neutralize ZIKV entry into mammalian cells. Moreover, immunization with prME‐PPSNs led to a robust cellular immune response with Th1 polarization. CD4^+^ and CD8^+^ T cell responses are known to be key mediators of protection against ZIKV infection, suggesting that the protection observed in the vaccinated mice might be a combination of neutralizing antibodies and T cell responses. Mice vaccinated with prME‐PPSNs exhibited no morbidity or mortality after a lethal ZIKV challenge, and the protective responses remained at a high level for 8 weeks, suggesting long‐term protection. These preclinical data highlight the potential of PPSNs‐based mRNA vaccines as promising candidates for clinical application.

Our PPSNs‐based platform offers distinct advantages compared to the platforms utilizing LNPs that have recently gained approval. First, PPSNs exhibit a highly desirable biodistribution pattern, with localized protein expression primarily at the injection site and negligible expression detected in other organs. In contrast, mice injected with FLuc‐LNPs displayed elevated luciferase expression levels in the liver and spleen, indicating systemic distribution. Second, the manufacturing process of mRNA‐PPSNs is notably simplified compared to mRNA‐LNPs, eliminating the need for a microfluidics device. The preparation of mRNA‐PPSN complexes involves a straightforward mixing step, allowing for convenient generation of the PPSNs‐based mRNA vaccine by combining nanoparticles and mRNA solution prior to injection. In contrast, the production of mRNA‐LNPs necessitates the use of specialized equipment. This simplicity in manufacturing holds significant potential for streamlining the production process of PPSNs‐based mRNA vaccines, potentially leading to cost reductions and improved accessibility. Furthermore, the immunogenicity of prME‐PPSNs proved superior to that of prME‐LNPs. Mice immunized with prME‐PPSNs exhibited significantly higher levels of neutralizing antibodies compared to those vaccinated with mRNA‐LNPs. Additionally, prME‐PPSNs immunization induced significantly higher levels of ZIKV E‐specific CD4^+^ and CD8^+^ T cell responses compared to prME‐LNPs vaccination. These factors collectively suggest that PPSNs can serve as a potential alternative to LNPs in the development of mRNA‐based vaccines for infectious diseases, highlighting the encouraging potential of PPSNs for mRNA vaccine development.

PPSNs demonstrated significant induction of anti‐ZIKV E IgG titers, high levels of neutralizing antibodies, and robust cellular responses even with a single injection, potentially due to the adjuvant effects of PPSNs. Silica nanoparticles have been reported to activate the NALP3 inflammasome, triggering the release of the bioactive form of the potent pro‐inflammatory cytokine IL‐1β.^[^ ^28^, ^29^, ^30^ ^]^ Additionally, the surface hydroxyl groups of PPSNs could induce complement activation through the alternative pathway.^[^ ^31^, ^32^ ^]^ We speculate that the activation of NALP3 and complement binding by PPSNs likely contribute to the observed immune responses.

In our study, we specifically targeted ZIKV, as it caused a pandemic in South America starting in 2015 and has been associated with neurological disorders such as congenital Zika syndrome and Guillain‐Barré syndrome. ZIKV is primarily transmitted by *Aedes* mosquitoes but can also be transmitted through sexual contact, blood transfusion, and maternal‐fetal transmission.^[^ ^33^ ^]^ Currently, there are no FDA‐approved vaccines or antiviral drugs for ZIKV infection. The ZIKV E protein is the primary target for neutralizing antibodies, as it mediates the virus's entry into host cells through interactions with cell surface receptors.^[^ ^34^, ^35^ ^]^ Our strategy involved intracellular expression of prM and E glycoproteins to induce the formation of virus‐like particles (VLPs). The assembly of virions takes place at the endoplasmic reticulum (ER) membrane in the last stages of the replication cycle of ZIKV, resulting in the formation of immature virions composed of icosahedrally arranged prME heterotrimers. Then, virions are transported to the *trans*‐Golgi network (TGN). The acidic environment of the TGN causes cleavage of prM by host protease furin, generating mature infectious virions. Therefore, intracellular expression of prM and E glycoproteins results in the formation of SVPs. The delivery of the ZIKV prME‐encoded gene has demonstrated protective effects against ZIKV in various animal models.^[^ ^30^, ^31^ ^]^

In the context of future clinical applications, our system presents several critical aspects that require careful consideration. Primarily, evaluating the thermostability of the mRNA‐PPSNs vaccine is essential. One of the significant challenges faced by mRNA vaccines lies in their limited thermostability, often necessitating ultra‐cold storage conditions. Overcoming this hurdle required the development of a thermostable vaccine with robust clinical efficacy. Additionally, further studies are needed on the incorporation of modified nucleosides into mRNA. In this study, we utilized unmodified mRNA as a delivery cargo to facilitate direct comparisons between the efficacies of PPSNs and LNPs. However, the use of modified nucleosides has been reported to mitigate the intrinsic immunostimulatory activity of exogenous mRNA and enhance its translation,^[^ ^1^, ^2^ ^]^ which may improve the clinical applicability of PPSNs‐based mRNA vaccine. It is also important to assess the vaccine's potential in protecting against maternal‐to‐fetal transmission because previous reports have highlighted the impact of ZIKV infections in fetuses from infected pregnant women, leading to congenital Zika syndromes such as microcephaly, congenital malformations, and fetal demise.^[^ ^36^ ^]^ Moreover, the duration of both humoral and cellular immune responses elicited by the prME‐PPSNs vaccine, along with further toxicity tests, should also be investigated in a non‐human primate model before considering its clinical application.

In conclusion, we developed a highly effective mRNA‐PPSNs vaccine platform against ZIKV infection. PPSNs serve as an efficient non‐viral delivery system, eliciting potent neutralizing antibodies and T cell responses. The safety profile of mRNA‐PPSNs was favorable, with mice exhibiting no side effects following immunization. The facile synthesis of both PPSNs and mRNA makes the PPSNs‐based mRNA vaccine platform well‐suited for the development and production of vaccines, particularly in response to emerging infectious diseases (EIDs) where a rapid response is crucial. Our findings contribute to the expanding field of mRNA‐based therapeutics and provide a promising avenue for future development of vaccines against ZIKV and other infectious diseases.

## 4 Experimental Section

### Materials

Tetramethyl orthosilicate (TMOS), toluene, dimethyl sulfoxide (DMSO), mesitylene (trimethyl benzene, TMB), and chloroform were purchased from Sigma‐Aldrich. Sodium hydroxide was purchased from Junsei Chemical Co., Ltd. and cetyl trimethyl ammonium bromide (CTAB) was obtained from Acros. ALC‐0315 and ALC‐0159 were obtained from MedChemExpress. 1,2‐distearoyl‐sn‐glycero‐3‐phosphocholine (18:0 PC, DSPC) was purchased from Avanti Polar Lipids. Cholesterol was obtained from Sigma‐Aldrich. 1.8k branched PEI was obtained from Polysciences. mRNA‐encoded FLuc was purchased from TriLink BioTechnologies. HiScribe T7 ARCA mRNA Kit (E2060S) was purchased from New England Biolabs. LIVE/DEAD fixable violet dead cell stain kit (L34955) was purchased from Invitrogen. APC/Cy7 anti‐mouse CD3 (100 221), PerCP anti‐mouse CD4 (100 431), PerCP anti‐mouse CD8 (100 731), FITC anti‐mouse IFN‐γ (505 806), and PE/Cy7 anti‐mouse TNF‐α (506 323), APC anti‐mouse IL‐2 (503 809) were purchased from Biolegend. Anti‐iFNAR1 blocking antibody (MAR1‐5A3) was obtained from Leinco. Goat anti‐mouse IgG HRP (31 430) was purchased from Invitrogen. Anti‐Mouse IgG‐FITC antibody produced in goat (F0257) was obtained from Sigma‐Aldrich.

### Viruses and Cells

ZIKV MR766 (VR‐1838) was purchased from the ATCC and ZIKV Brazil/16 321 was purchased from the Virus Research and Testing Center, Korea. MR766 was a mouse‐adapted strain that has been described previously.^[^ ^30^ ^]^ Virus stocks were propagated in the Vero E6 cells with DMEM supplemented with 2% FBS and P/S and titrated by focus forming assay (FFA), as described previously.^[^ ^37^ ^]^ Vero E6 cells were maintained in DMEM supplemented with 10% FBS and P/S. C2C12 cells were maintained in DMEM supplemented with 10% heat‐inactivated FBS and P/S.

### Mouse Experiments

This study was conducted following the guidelines of the recommendations from the Association for Assessment and Accreditation of Laboratory Animal Care. All animal experiments were approved by the Institutional Animal Care and Use Committees (IACUC) of Seoul National University, South Korea (SNU‐220623‐5). C57BL/6 male mice (6‐week old) were purchased from NARA Biotech (Korea), and had an acclimatization period for 2 weeks. All mice were housed in pathogen‐free mouse facilities. For vaccination, mice were intramuscularly injected with 30 µL of buffer or prME‐PPSNs (prME mRNA, 15 µg; PPSNs, 30 µg).

For ZIKV challenge experiments, intraperitoneal injection of 2 mg of anti‐iFNAR1 blocking antibody was given 24 h prior to viral infection. Mice were inoculated subcutaneously in the rear footpad with 10^3^ FFU of African genotype ZIKV MR 766 or 10^5^ FFU of Asian genotype ZIKV Brazil/16 321. Mice were monitored for weight loss, mortality, and clinical score (1: healthy; 2: slightly ruffled fur; 3: ruffled fur; 4: very ruffled fur, walking, but no scurrying; 5: very sick, slow to no movement; 6: moribund requiring humane euthanasia; 7: deceased), as previously reported.^[^ ^38^ ^]^

### PPSN Synthesis and Characterization

To prepare bare PSNs, the previously reported method was used.^[^ ^22^ ^]^ Briefly, MeOH/DW (0.6/0.4 = w/w) were mixed with CTAB and 1 M NaOH solution. Then, TMOS was added to the mixture at room temperature. The mixture was vigorously stirred for 8 h and then aged overnight. After five washes with ethanol and water, the resultant white precipitate was purified to eliminate any leftover surfactant. Following sonication of as‐produced silica nanoparticles in EtOH for 30 min, a 1:1 (v/v) solution of water and TMB were added to the mixture. The mixture was then kept at 140 °C for four days. Five times each ethanol and water were used to wash the resultant white powder. DW containing PEI was mixed with nanoparticles and the mixture was then agitated for 4 h at room temperature. The particles were centrifuged and washed. For the TEM‐EDS investigation, JEM‐F200 (JEOL Ltd.) at the National Center for Inter‐university Research Facilities (NCIRF) at Seoul National University was used. The Nova 2000e (Quantachrome) was used to measure BET surface area and pore size and Zetasizer Nano ZS (Malvern) determined the hydrodynamic diameter and zeta potential.

### In Vitro Cellular Uptake

C2C12 cells were seeded in a 24‐well plate containing 0.5 mL of DMEM supplemented with 10% heat‐inactivated FBS and P/S. After 24 h, the cells were pretreated with chlorpromazine (5 µg mL^−1^) to inhibit clathrin‐dependent endocytosis, genistein (200 µM) to inhibit caveolae‐dependent endocytosis, nocodazole (20 µM) to inhibit macropinocytosis, or incubated at 4 °C for 1 h in serum‐free media. Following pretreatment, dye‐labeled mRNA‐PPSNs were added and incubated for an additional hour. The cells were then collected by centrifugation, washed with PBS, and analyzed using the BD FACSLyricTM (BD, USA) system. To quantify the relative uptake efficiency, the median fluorescence intensity (MFI) of the cells were measured. The MFI values of the cells were normalized to the MFI of the control group, which was incubated at 37 °C without any endocytosis inhibitors.

### LNP Formulation

A lipid combination composed of ALC‐0315/ALC‐0159/DSPC/Cholesterol with mole ratios of 46.3/9.4/42.7/1.6 was prepared to encapsulate mRNA into LNPs. Each lipid was mixed in ethanol and mRNA solution was prepared in sodium acetate buffer to reach 1 mg mL^−1^. The organic and aqueous phases were then combined using Spark NanoAssmblr (Precision NanoSystems) with constant flow rate and the final molar N/P ratio was 6. After that, A Slide‐A‐Lyzer Dialysis Cassette (MWCO, 10 KDa) was used to dialyze LNPs overnight against PBS (pH 7.4).

### RNase Protection Assay

Equal amounts of mRNA (0.5 µg) were loaded onto PPSNs (1 µg) or naïve PEI (1 µg) in a 10 µL PBS solution. Following the addition of RNase (Sigma‐Aldrich) at a final concentration of 0.05%, the samples were incubated at 37 °C for various durations. Subsequently, the samples were treated with heparin and analyzed using a gel retardation assay.

### In Vitro Transcription

The manufacturer's instructions were followed for in vitro transcription. The pMRNAxp mRNAExpress Vector (System Biosciences) was used for cloning the codon‐optimized ZIKV prME glycoproteins of ZIKV SPH/2015 containing the signal sequence from the JEV. The pMRNAxp mRNAExpress Vector includes a 5′ UTR, 3′ UTR, and a polyA tail. ZIKV prME mRNA was transcribed using HiScribe T7 ARCA mRNA Kit with unmodified nucleosides (New England Biolabs).

### Western Blot

HEK293T cells were lysed with RIPA buffer (Thermo Scientific) according to the manufacturer's instructions. The protein samples were separated on SDS‐PAGE gel and transferred to polyvinylidene difluoride membrane. The membrane was blocked for 1 h in PBS buffer containing 0.2% Tween 20 (PBST) with 5% skim milk. The membrane was washed 3 times in PBST and incubated in PBST with 5% skim milk and 1:1000 primary antibody (pan‐flavivirus E‐specific monoclonal antibody 4G2) overnight at 4 °C. After 3 washes in PBST, the membrane was incubated with 1:10 000 secondary antibody (Rabbit Anti‐Mouse IgG H&L (HRP)) in PBST containing 5% skim milk for 4 h at 4 °C. After 3 washes in PBST, the membrane was imaged using a Chemidoc MP imaging system (BioRad).

ZIKV E protein in the supernatant of HEK293T cells was pelleted by ultracentrifugation. Supernatants of HEK293T cells were harvested and cleared by centrifugation at 3,000 rpm for 15 min and 10,000 rpm for 30 min. Clarified supernatants were then concentrated by ultracentrifugation at 30,000 rpm for 2.5 h. Pellets were then resuspended in PBS. The samples were then subjected to a western blot.

### Immunofluorescence Staining

FLuc mRNA or prME mRNA was transfected into Huh‐7 cells in 24‐well plates. To serve as controls, non‐transfected cells were included as a negative control, and ZIKV‐transfected cells were included as a positive control. At 24 h post‐transfection or infection, the cells were washed with PBS and fixed with 4% paraformaldehyde. The fixed cells were permeabilized and blocked with a solution containing 10% FBS and 10% BSA in PBS for 1 h. The cells were then incubated with a pan‐flavivirus E‐specific monoclonal antibody 4G2, followed by FITC‐conjugated anti‐mouse secondary antibody. Nuclei were stained with Hoechst 33 342. Finally, the stained cells were observed under a fluorescence microscope (IX71, Olympus) to visualize the cytoplasmic expression of the ZIKV E protein.

### Bioluminescence Imaging

Bioluminescence imaging was performed using the IVIS Spectrum In Vivo Imaging System (PerkinElmer) to detect luciferase activity. Mice were intramuscularly injected with FLuc mRNA encapsulated in either PPSNs or LNPs. For in vivo bioluminescence imaging, mice were intraperitoneally administered 150 mg kg^−1^ of D‐Luciferin, Potassium Salt (GoldBio), and luminescent images were captured five minutes later. Regions of interest (ROIs) were selected using Living Image Software (PerkinElmer), and the total flux was measured.

For ex vivo imaging, mice were intramuscularly injected with FLuc mRNA encapsulated in either PPSNs or LNPs. At 6 h post‐injection, mice were intraperitoneally administered 150 mg kg^−1^ of D‐Luciferin, Potassium Salt (GoldBio). Immediately after necropsy, tissues of interest were individually placed in wells of a 24‐well plate. Sufficient 300 µg mL^−1^ of D‐Luciferin was added to cover the tissues, and luminescent images were acquired after five minutes.

### Intracellular Cytokine Staining

Intracellular cytokine staining was conducted as previously described.^[^ ^30^ ^]^ Spleens were collected and teased. Splenocytes were washed in a complete medium and centrifuged for 10 min at 4 °C. Cells were washed with PBS and counted with LUNA‐II cell counter (Logos Biosystems). Splenocytes (2 × 10^6^ cells well^−1^) were stimulated in a 96‐well cell culture plate using 2 µg mL^−1^ recombinant ZIKV E protein (Sino Biological) for 6 h at 37 °C, 5% CO_2_. 20 µl of GolgiPlug (brefeldin A, 1:100, BD Biosciences) and GolgiStop (monensin, 1:143, BD Biosciences) were added to each sample during the last 5 h of incubation. Following centrifugation at 4 °C for 10 min, cells were with LIVE/DEAD fixable violet dead cell stain kit (Invitrogen) following the manufacturer's instructions. Cells were washed twice and surface stained for CD3, CD4 and CD8. Following surface staining, cells were fixed and permeabilized using BD Cytofix/Cytoperm kit (BD Biosciences) according to the manufacturer's instructions. Finally, intracellular cytokine staining for induction of IFN‐γ, TNF‐α, and IL‐2 was done at 4 °C for 1 h. Subsequently, cells were washed twice and resuspended in PBS with 0.5% BSA. Cells were analyzed on BD FACSLyric (BD Biosciences). The flow cytometry gating strategy presented in Figure S12 (Supporting Information) was employed.

### ELISpot Assay

Harvested spleens were dissociated using gentleMACS Dissociator (Miltenyi Biotec) and filtered through 70 µm cell strainer. Cells were treated with 1× Red Blood Cell Lysis Solution (Miltenyi Biotec). After RBC removal, cells were washed and resuspended in RPMI with 10% FBS with a concentration of 1.0 × 10^7^ cells/mL cells. 2.0 × 10^5^ cells per well was added and incubated overnight in a cell incubator for cell resting. After the resting, cells were stimulated by 10 µg m^−1^L ZIKV E protein for 24 h, and IFN‐γ and IL‐4 secretion were detected by FluoroSpot Plus (Mabtech) following the manufacturer's instructions. The spot‐forming cells (SFCs) were manually counted using inverted microscope (IX71, Olympus).

### ELISA

Immunoplate (SPL Life Sciences) was coated with 6 µg mL^−1^ recombinant ZIKV E protein (Sino Biological). The plate was blocked with blocking buffer (2% BSA in PBS) and washed with wash buffer (PBS with 0.05% Tween 20). Mouse serum was serially diluted and incubated on Immunoplate for 1 h at room temperature. After incubation, the plate was washed 3 times using wash buffer. Anti‐mouse IgG‐HRP was added to the plate and incubated for 1 h. After 3 times washing with wash buffer, the plate was added with TMB substrate and 2N sulfuric acid was used to stop the process. The absorbance at 450 nm was monitored by SynergyMx (Biotek). To determine the ZIKV E‐protein‐specific IgG endpoint dilution titer, the highest reciprocal dilution of serum was used with an OD value above 2 times the average OD of the background.

### Focus Reduction Neutralization test (FRNT)

Serum samples were collected on day 14, 35, and 56. Heat‐inactivated mouse serum was serially diluted and mixed with ZIKV (Brazil/16 321) for 1 h at 37 °C. Serum‐virus mixtures were incubated with Vero E6 monolayers in a 96‐well cell culture plate for 1 h at 37 °C. Then, FFA was performed as described previously.^[^ ^37^ ^]^

### qRT‐PCR

Total RNA was isolated using Trizol (Invitrogen) from mouse serum collected 3 or 7 days after the challenge according to the manufacturer's instructions. The cDNA samples were prepared from 0.1 to 1 µg of total RNA using reverse transcription using SuperScript II Reverse Transcriptase (Invitrogen). Synthesized cDNA samples were subjected to qRT‐PCR with primers previously described (NS5‐2362F: 5′‐GACTGGGTTCCAACTGGGAG‐3′; and NS5‐2457R: 5′‐ CCACACTCTGTTCCACACCA‐3′).^[^ ^39^ ^]^ Real‐time PCR was conducted to quantify viral RNA in a 20 µL reaction volume with 10 to 100 ng of cDNA and 0.2 µM of each primer using a POWER SYBR Green Master Mix (Applied Biosystems Inc.) on a CFX Connect (Bio‐Rad). The two‐step amplification process involved annealing/extension at 60 °C for 1 min and denaturation at 95 °C for 15 s, followed by melting curve analysis. The resulting data were analyzed using Bio‐Rad CFX Maestro software, and the Cq value was automatically determined. To analyze viremia, a linear correlative standard curve was established between the log of the virus titer (FFU equivalents mL^−1^) and the corresponding Cq value. Each standard was tested in triplicate, and all were detectable by the assay. A standard curve was derived against a serially diluted viral titer.

### Statistical Analysis

GraphPad Prism 7 software was used to perform all statistical analyses as indicated in the figure captions. All values were presented as mean with SD or SEM.

## Conflict of Interest

Lemonex Inc. holds patents related to the porous nanoparticle and its use as drug delivery systems.

## Author Contributions

H.S., C.W. and D.‐H.M. performed conceptualization. H.S. and S.K. performed investigation. H.S. and D.‐H.M. performed formal analysis. H.S. performed visualization. H.S. wrote – original draft. H.S., S.K., C.W., and D.‐H.M. wrote – review and edited the original draft. D.‐H.M. performed Supervision.

## Supporting information

Supporting Information

## Data Availability

The data that support the findings of this study are available in the supplementary material of this article.
