# Supplementary material for: A Single‐Dose mRNA Vaccine Employing Porous Silica Nanoparticles Induces Robust Immune Responses Against the Zika Virus
Source: Adv Sci (Weinh). 2024 Jul 15;11(35):2404590. doi: 10.1002/advs.202404590 (PMC11425238; doi:10.1002/advs.202404590)
Supplement: Supplementary file 1 — Supporting Information [file ADVS-11-2404590-s001.docx]

Supporting Information

A single-dose mRNA vaccine employing porous silica nanoparticles induces robust immune responses against the Zika virus

Hojeong Shin, Seounghun Kang, Cheolhee Won, and Dal-Hee Min*


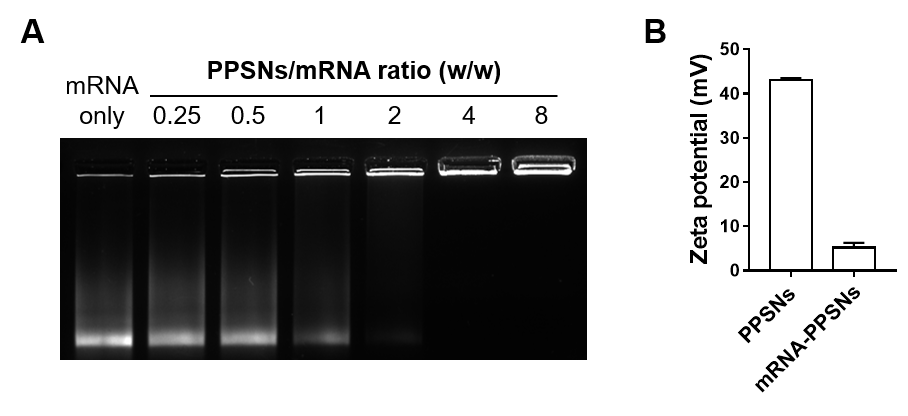


**Figure S1.** A) mRNA encapsulation efficiency of PPSNs. B) Change in zeta potential of PPSNs with mRNA encapsulation.


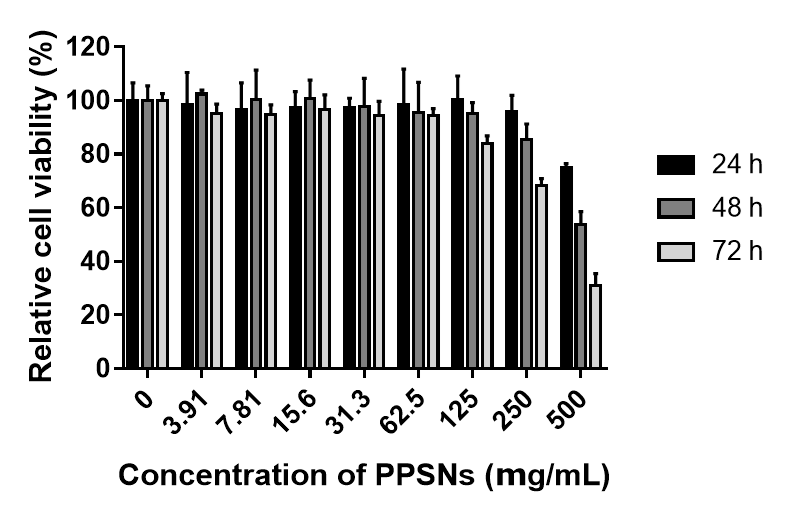


**Figure S2.** Relative cell viability of C2C12 cells treated with different concentrations of PPSNs. Data are presented as mean with SD (*n* = 4).


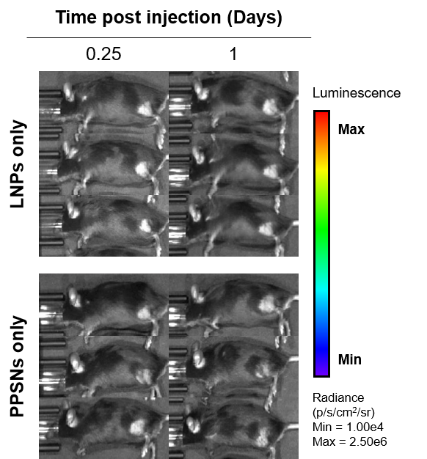


**Figure S3.** Representative IVIS images of C57BL/6 mice injected intramuscularly LNPs only or PPSNs only, without mRNA payload.


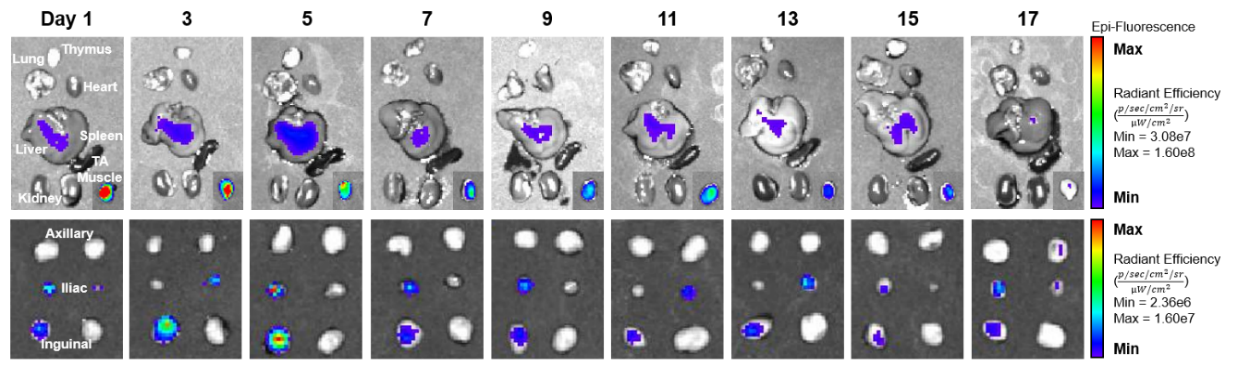


**Figure S4.** Fluorescent images of major organs and lymph nodes at different time points after the injection of dye-conjugated mRNA-PPSNs.


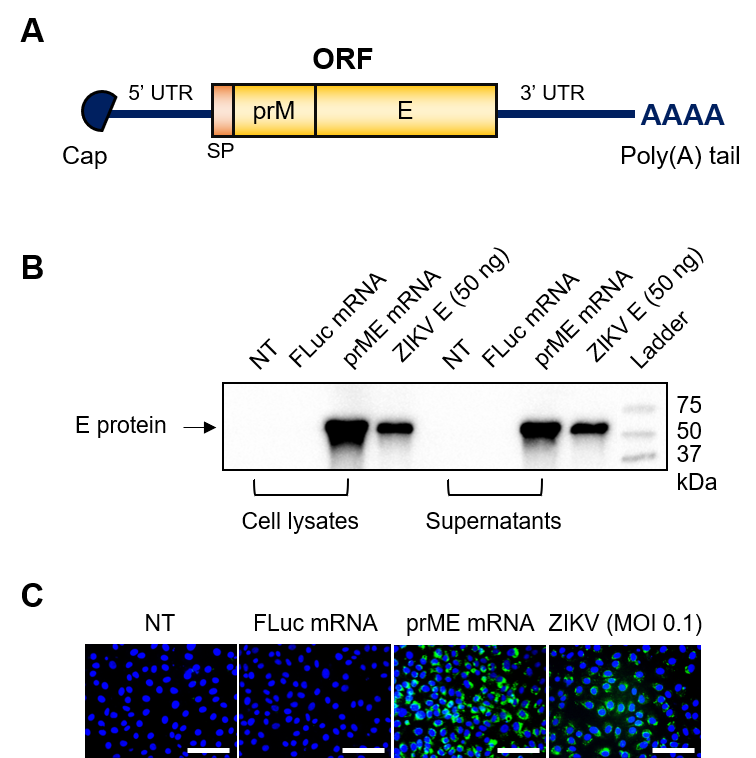


**Figure S5.** Characterization of ZIKV prME mRNA expression in mammalian cells. (A) The ZIKV prME mRNA encodes the signal peptide (SP) from Japanese encephalitis virus (JEV) and pre-membrane (prM) and envelope (E) glycoproteins from ZIKV SPH/2015. (B) HEK293T cells were transfected with ZIKV prME mRNA. Cell lysates and supernatants were subjected to western blot analysis using the 4G2 antibody specific to flavivirus E protein. ZIKV E protein (50 ng) was used as a positive control. Representative results from at least two experiments are shown. (C) Immunofluorescence staining of ZIKV E protein expression in Huh-7 cells, observed using fluorescence microscopy. The images depict the non-transfected control (NT), FLuc mRNA-transfected cells, prME mRNA-transfected cells, and ZIKV-infected cells. The scale bar represents 100 μm.


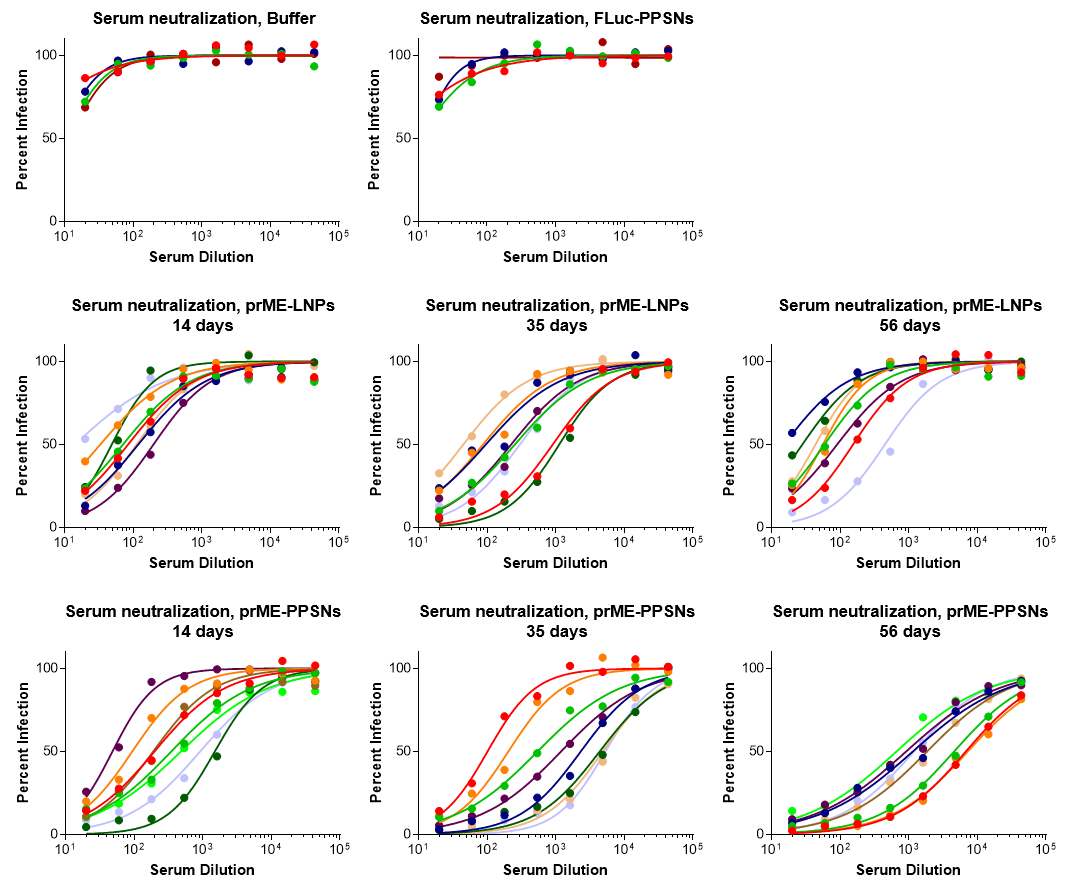


**Figure S6.** Serum neutralization curves from C57BL/6 mice vaccinated with buffer, FLuc-PPSNs, prME-LNPs, and prME-PPSNs, related to Figure 4B.


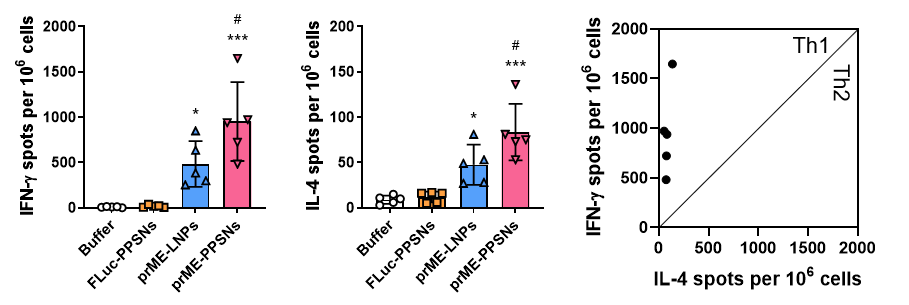


**Figure S7.** Induction of Th1-biased T cell responses by prME-PPSNs vaccination. C57BL/6 mice were intramuscularly immunized with buffer, FLuc-PPSNs, prME-LNPs, and prME-PPSNs (*n* = 5). On day 28, splenocytes were harvested and stimulated with ZIKV E protein for a direct *ex vivo* IFNγ and IL-4 ELISpot assay. Data are presented as mean with SD. Statistical significance was determined using one-way ANOVA. Asterisks (**P* < 0.05 and ****P* < 0.001) indicate the comparison of prME-LNPs and prME-PPSNs to the buffer group. The differences between prME-LNPs and prME-PPSNs are indicated by hashtags (^#^*P* < 0.05). Not-significant p-values are not indicated.


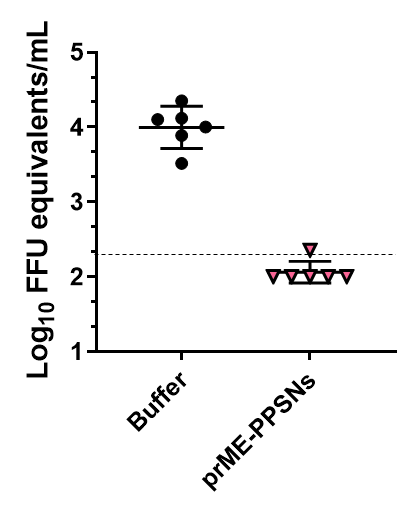


**Figure S8.** Single immunization of ZIKV prME-PPSNs provides protection against Asian genotype ZIKV Brazil/16321. C57BL/6 mice were immunized with buffer or ZIKV prME mRNA-PPSNs (*n* = 6). At week 8, vaccinated C57BL/6 mice received 2 mg of anti-INFAR-1 blocking antibody, and 1 day later, they were challenged with 10^5^ FFU of ZIKV. Serum was collected 3 days after viral challenge and analyzed for ZIKV RNA levels. Data are presented as mean with SD. Dotted line indicates the limit of detection.


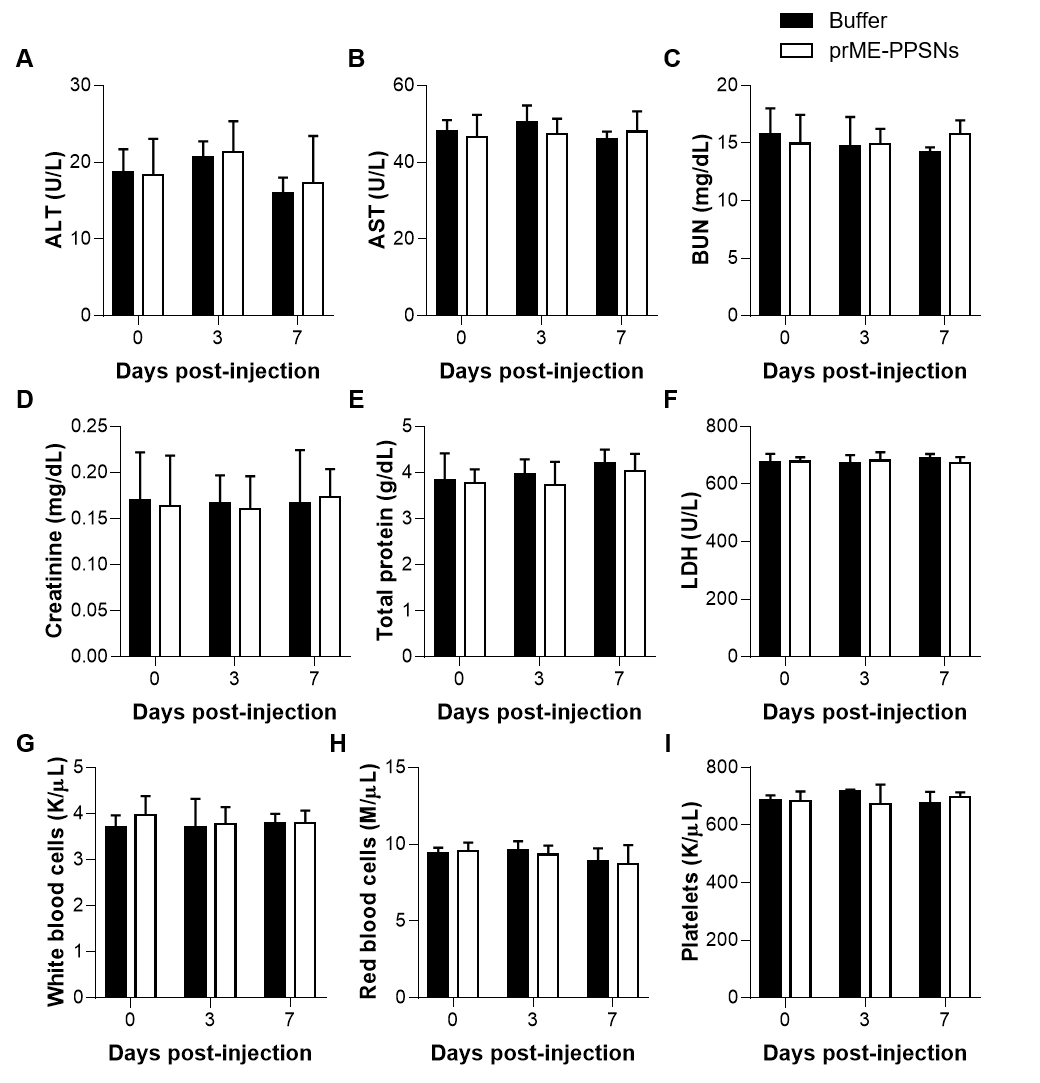


**Figure S9.** Toxicological assessment of prME-PPSNs. Serum concentrations of (A) alanine aminotransferase (ALT), (B) aspartate aminotransferase (AST), (C) blood urea nitrogen (BUN), (D) creatinine, (E) total protein, and (F) lactate dehydrogenase (LDH) were measured after vaccination with buffer or prME-PPSNs. Hemocytometer results for (G) white blood cell counts, (H) red blood cell counts, and (I) platelet counts showed no blood cell toxicity. Data are presented as mean with SD (*n* = 3).


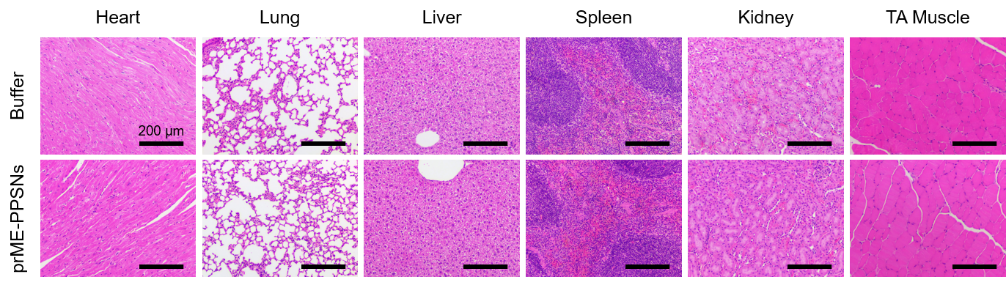


**Figure S10.** Histopatholoical analysis of major organs after vaccination. Representative images of H&E stained organs harvested from each group of mice at 15 day after immunization.


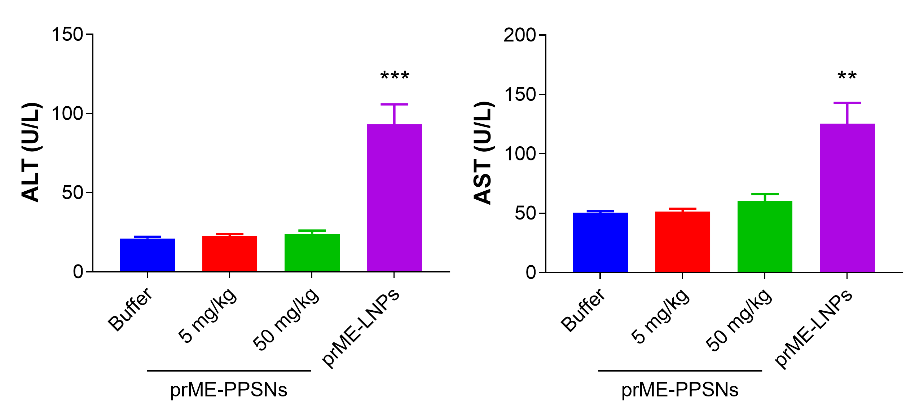


**Figure S11.** Measurement of liver toxicity markers ALT and AST in serum collected 3 days after administration of prME-PPSNs (5 and 50 mg/kg) or prME-LNPs (5 mg/kg). Data are presented as mean with SD (*n* = 3). Statistical significance was determined using one-way ANOVA. ***P* < 0.01 and ****P* < 0.001; not-significant p-values are not indicated.


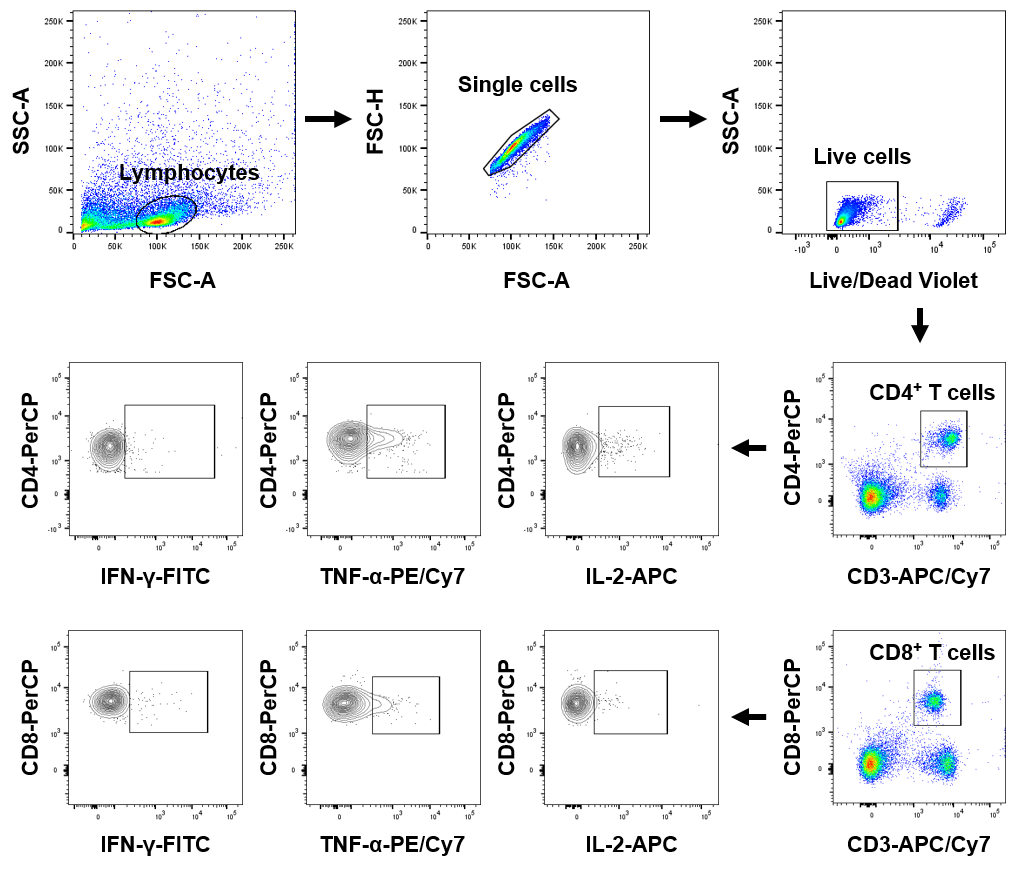


**Figure S12.** Gating strategy for intracellular cytokine staining.
